# Oral health-related quality of life and early childhood caries among preschool children in Trinidad

**DOI:** 10.1186/s12903-016-0324-7

**Published:** 2016-12-07

**Authors:** Rahul Naidu, June Nunn, Erica Donnelly-Swift

**Affiliations:** 1 School of Dentistry, The University of the West Indies, Saint Augustine, Trinidad and Tobago; 2 School of Dental Sciences, Trinity College Dublin, Dublin 2, Ireland

**Keywords:** Early childhood caries, Quality of life, Preschool children, Caribbean

## Abstract

**Background:**

Early childhood caries (ECC) is a public health problem in developed and developing countries. The purpose of this study was to describe the relationship between oral health-related quality of life (OHRQoL) and ECC among preschool children in a Caribbean population.

**Method:**

Parents/primary caregivers of children attending nine, randomly selected preschools in central Trinidad were invited to complete an oral health questionnaire and have their child undertake an oral examination. The questionnaire included the Early Childhood Oral Health Impact Scale (ECOHIS). Visible caries experience was assessed using WHO criteria. Logistic regression models were used to determine the factors associated with OHRQoL and ECC.

**Results:**

Three hundred nine parents/caregivers participated in the study (age-range 25–44 years) and 251 children (mean age 3.7 years) completed oral examinations. Adjusting for other factors, the odds for a child aged 4 years of having dental caries were greater than the odds for a child aged 3 years (OR 3.61; 95% CI (1.76, 6.83). The odds for children having difficulty drinking hot or cold drinks were greater for those with dental caries than the odds for children who have no such difficulty. Similarly, the odds for children who had difficulty eating were greater for those with dental caries than the odds ratios for children who had no difficulty eating (OR 8.29; 95% CI (2.00, 43.49). Adjusting for the effects of other factors, the odds of parents/caregivers feeling guilty were greater if their child had experienced dental caries in comparison to parents/caregivers whose child did not have dental caries (OR 3.50; 95% CI (1.32, 9.60). Adjusting for other factors, the odds of parents/primary caregivers having poor quality of life was increased when they had a child with a dmft in the range 1–3 (OR 2.68; 95% CI (1.30, 5.64) dmft > 4 (OR 8.58; 95%CI (3.71, 22.45), in comparison to those whose child had a dmft = 0.

**Conclusion:**

In this sample of preschool children OHRQoL was associated with ECC. More negative impacts were found in children with a greater severity of visible caries experience. This suggests the need for strategies to prevent and manage ECC in this Caribbean population.

## Background

Early Childhood Caries (ECC) has been defined by the American Academy of Pediatric Dentistry as ‘the presence of one or more decayed, missing due to caries, or filled tooth surfaces in any primary teeth in children under 6 years of age [1]. In children younger than 3 years of age, any sign of smooth-surface caries is indicative of severe early childhood caries (S-ECC) [1].

Beyond the immediate distress caused by toothache, early childhood caries (ECC) can also have longer term negative, health outcomes [2, 3]. Untreated decay in infancy and early childhood is believed to affect weight gain and overall growth and development [4]. Along with these patho-physiological effects, ECC can impact on oral health related quality of life (OHRQoL) [2, 3]. As parents and caregivers have the main responsibility for their preschool-aged children, ECC can also affect them indirectly, for example, work-loss and financial impact due to having to stay at home to take care of the child [5]. ECC is therefore recognized as a public health problem due to its high prevalence in some populations and the potential for negative health impacts if left untreated [6, 7].

The few studies that have been undertaken in the English-speaking Caribbean suggest that caries prevalence among infants and preschool children in the region is high [8, 9]. In central Trinidad, the prevalence of ECC among 251 preschool children was reported as 29.1% with the majority of this being untreated, decayed teeth and 12% of children were in need of urgent care or referral [10]. Affordability and access to dental care for people from lower socioeconomic groups and those living in rural locations is a challenge in Trinidad and Tobago as most of the county’s registered dentists work in private practices, generally clustered in urban centers.

Although there are international reports on OHRQoL of preschool children [11–16], nothing is known about the effect of ECC on OHRQoL among preschool children in the Caribbean.

Understanding the impact of dental caries in young children and their families can guide the development of treatment and preventive protocols as part of dental service planning.

The aim of this study was to describe the relationship between OHRQoL and ECC among preschool children in Trinidad.

## Method

A cross-sectional oral health survey of preschool children was undertaken in the Caroni region of central Trinidad. The accessible population were children aged 3 to 5 years of age, attending preschools in the Caroni Education District. Based on the list of registered preschools, there were 27 government/government-assisted and 57 non-government preschools in the district at the time of the survey, with an enrolled population of approximately 2000 children. Previous data from Anguilla [9] (which estimated prevalence at 30%) was used to determine that 340 children were required to assess caries prevalence within the preschool population in the district. This figure accounted for 6% precision and 20% non-response rate.

Sampling consisted of cluster sampling within the Caroni Education District. A total of ten schools were selected by systematic random sampling from the school lists (three government/assisted schools and seven non-government preschools). Each cluster consisted of all registered children within the preschool. Each preschool was assumed to have an average of 30 registered children. Very small schools (<15) and very large schools (>60) were excluded, in order to enable inclusion of preschools of similar sizes and enable data collection by a single examiner. Stratification was not employed.

Ethical approval for the study was obtained from The University of the West Indies, Faculty of Medical Sciences Research Ethics Committee. Permission for the selected preschool’s inclusion in the study was obtained from individual head teachers and written positive consent was requested from parents and caregivers for the oral examinations. Self-administered oral health questionnaires were provided to participating preschools. These questionnaires were then given to all parents and caregivers by the school administration, along with a consent form.

OHRQoL was measured using the Early Childhood Oral Health Impact Scale (ECOHIS) [3], included as part of the oral health questionnaire. The ECOHIS is a short, condition-specific tool, to be completed by the child’s parent or primary caregiver. It has been validated in the English language and translated versions are reported to have good psychometric properties [3]. The ECOHIS consists of questions relating to quality of life domains for both the child and the family. These domains include: symptoms, function, psychological effects, self-image, parent distress and family function [3]. Responses are based on the scale: *Never*, *Hardly ever*, *Occasionally*, *Often*, *Very often*, *Don’t know*. Scores for the instrument are calculated from the sum of responses for the child (0–36) and family sections (0–16) and reported as mean impacts per item/section (maximum overall score of 52). Higher mean ECOHIS score represents worse OHRQoL.

The ECOHIS instrument was piloted among 30 parents and caregivers of young children attending a dental hospital clinic in Trinidad. The instrument was found to have acceptable face and content validity and thus did not require any modification. The ‘don’t know’ response were treated as ‘missing’ for the analyses.

Dental examinations were undertaken by a single, trained and calibrated dentist (RN) using WHO criteria [17]. Training and calibration was achieved by use of clinical slides on CD ROM, representing all categories of caries to be assessed and recorded. This was done under the supervision of a dental epidemiologist (JN). Examinations took place in classrooms using natural light, with the child in a seated position on a small chair/bench with the examiner positioned behind. Teeth were assessed visually with the use of a disposable mouth mirror, with the examiner wearing disposable gloves and facemask. New gloves and a mouth mirror were used for each child. Teeth were not air dried but soft debris on tooth surfaces was removed with a cotton roll or gauze square.

Examiner reliability was assessed by re-examination of children at one preschool (25 children). These re-examinations took place on the same day as data collection. The Kappa statistic for intra-examiner reliability was 0.9. Data collection was undertaken over a three-month period.

In the field, oral examination data were entered onto a record sheet by a research assistant. This information was subsequently transferred to a computer database (SPSS v 16) for storage and processing. Data were cleaned and checked for transcription errors before processing using SPSS version 16 for Windows and STATA version 10.

### Statistical analysis

Logistic regression models were adopted to determine the family and child related factors associated with dmft and ECOHIS. Specifically, models examining factors relating to dmft were used to examine the odds of children having a dmft > 0 compared to the odds of children with a dmft = 0. Similarly, the odds of a parent/primary caregiver having an ECOHIS score >0 was compared to the odds of parents/primary caregivers with an ECOHIS score = 0, taking sociodemographic factors into account. Child and/or parent-primary caregiver characteristics were included as fixed effects in the models and a random intercept was included to account for cluster variation (i.e. variation within preschools). Akaike’s information criteria and likelihood ratio tests were used to evaluate goodness of fit. In addition to variables retained in the final model, all models were adjusted for age and sex. Model sensitivity and specificity were examined using receiver operating characteristic curves and area under the curve (AUC). If a model achieves perfect sensitivity and specificity, then the AUC would have a value of 1. If the AUC has a value of 0.5 then the model achieves poor sensitivity and specificity. Despite estimation of AUC, the models developed for this research are for descriptive purposes, they are not intended for prediction. Generalised variance inflation factors (GVIF) and adjusted GVIF were used to determine the presence of multicollinearity. Model results are displayed in terms of odds ratios (ORs) and corresponding 95% confidence interval (CI). ORs have a range from 0 to infinity. An OR equal to one, denotes that there is no difference in odds whereas an OR greater than 1 indicates, for instance, that the ratio of those with a dmft > 0 versus a dmft = 0 in the selected group is greater than the baseline group. If there is no evidence to suggest that the ratio of those with dmft > 0 (versus dmft = 0) for the selected group are different from the baseline group, then the 95% CI for the OR will contain 1 in the interval.

Statistical analysis was performed using statistical software R (version 3.2.3) [18].

## Results

From an enrolment of 340 children, 314 parents gave consent for the oral examination (91% response rate). Of these children, 36 (11.5%) were absent on the day and 27 (8.6%) refused examination. Three hundred and nine parents completed the questionnaire (Table 1). The mean age of the parents and primary caregivers was not determined as the questionnaire only recorded respondent age-range. Among these 309 respondents, 90% of parents/primary caregivers were in the age range 25–44 years. Parent/primary caregiver ethnicity was 74.4% Indian, 11.3% African, 13.3% mixed and 1% white or other (Table 1).

**Table 1:** Socio-demographic information for all parents and caregivers (*N* = 309)

	n	Percent
Age group		
18–24	17	5.5
25–34	183	59.2
35–44	95	30.7
45–54	8	2.6
55–64	2	0.7
65+	1	0.3
Missing	3	1.0
Ethnic group		
African	35	11.3
Indian	230	74.4
Mixed	41	13.3
White	1	0.3
Other	2	0.7
Occupation		
Professional	11	3.6
Managerial/lower professional	48	15.5
Non-manual	44	14.2
Manual -skilled	107	34.6
Manual- semi-skilled	6	1.9
Manual –unskilled	43	13.9
Housewife/unemployed	15	4.9
Retired/old-age pensioner	7	2.3
Missing	28	9.1
Education		
None	1	0.3
Primary	29	9.4
Secondary	255	50.2
Technical college	54	17.5
University	49	15.9
Other	15	4.9
Missing	6	1.9

The ECOHIS showed good internal consistency with a Cronbach alpha reliability coefficient of 0.94. For the child and family sections Cronbach alpha was 0.92 and 0.85, respectively.

Overall, quality of life impacts were low, with median score being 0. Mean impacts scores for the whole instrument were 2.29 (sd 5.52) and for the child and family sections 1.09 (sd 3.62) and 0.80 (sd 2.16), respectively. Examination of responses relating to the child’s quality of life indicated that approximately 10.4% (32/309) reported that their child experienced pain in the teeth, mouth or jaw.

Approximately 5.2% (16/309) and 4.2% (13/309) reported that their child experienced difficulty eating some foods or difficulty drinking hot or cold drinks.

Examination of responses relating to family function indicated that approximately 10% (31/309) of parents/primary caregivers reported that they felt guilty, 5.2% (16/309) had been upset and 4.9% (15/309) reported that they had taken time off work due to their child’s oral health problems.

Despite 309 parent/primary caregivers completing the questionnaire, approximately 18% (58/309) of children did not complete the oral examination, thus a total of 251 children completed the oral examination. Of these children, 50.2% were male, with an age range of 3 to 5 years-old and mean age of 3.7 years (sd 0.67). Full results for visible caries experience have been reported previously [10]. Socio-demographic characteristic for parents/primary caregivers, together with information on ECOHIS and dmft for those children who completed the oral health assessment, are shown in Table 2.

**Table 2:** Socio-demographic information for parents/caregivers whose child completed the oral health assessment (*N* = 251)

	ECOHIS = 0		ECOHIS > 0		dmft = 0		dmft > 0	
	Count	%	Count	%	Count	%	Count	%
Parent/primary caregiver characteristics								
Age								
< 25 years	8	3.19	7	2.79	12	4.78	3	1.20
25–34	83	33.07	64	25.50	102	40.64	45	17.93
35–44	46	18.33	32	12.75	54	21.51	24	9.56
45+	7	2.79	2	0.80	8	3.19	1	0.40
Unknown	2	0.80	0	0.00	2	0.80	0	0.00
Sex								
Male	16	6.37	14	5.58	24	9.56	6	2.39
Female	130	51.79	91	36.25	154	61.35	67	26.69
Education								
Primary or lower	13	5.18	11	4.38	15	5.98	9	3.59
Secondary	47	18.73	38	15.14	85	33.86	40	15.94
Third level	74	29.48	51	20.32	64	25.50	21	8.37
Other	9	3.59	4	1.59	11	4.38	2	0.80
Unknown	3	1.20	1	0.40	3	1.20	1	0.40
Visits to the dentist								
Never	27	10.76	19	7.57	30	11.95	16	6.37
1–2 per year	82	32.67	55	21.91	101	40.24	36	14.34
Only when in pain	23	9.16	14	5.58	25	9.96	12	4.78
Other	14	5.58	16	6.37	22	8.76	8	3.19
Unknown	0	0.00	1	0.40	0	0.00	1	0.40
Child characteristics								
Age (years)								
3	56	22.31	29	11.55	71	28.29	14	5.58
4	74	29.48	64	25.50	87	34.66	51	20.32
5	16	6.37	12	4.78	20	7.97	8	3.19
Sex								
Male	70	27.89	56	22.31	89	35.46	37	14.74
Female	76	30.28	49	19.52	89	35.46	36	14.34
DMFT								
0	122	48.61	56	22.31				
1–3	16	6.37	20	7.97				
> 4	8	3.19	29	11.55				

Table 3 shows the frequency of oral health impacts for children with some caries experience (dmft > 0) and for those with no caries experience (dmft = 0) for the child and family levels, respectively.

**Table 3:** Oral health impacts for children with no visible caries dmft = 0 and some visible caries dmft > 0

	Never/hardly ever				Occasionally/often/very often				Don’t know/NA			
	dmft = 0		dmft > 0		dmft = 0		dmft > 0		dmft = 0		dmft > 0	
	n	%	n	%	n	%	n	%	n	%	n	%
Child impacts												
Pain in the teeth, mouth or jaw	170	76.9	51	23.1	6	22.2	21	77.8	2	66.6	1	33.3
Difficulty drink hot or cold drinks	174	73.7	62	26.3	2	18.2	9	81.8	2	50.0	2	50.0
Difficulty eating some foods	175	74.5	60	25.5	3	20.0	12	80.0	0	0	1	100
Difficulty pronouncing some words	175	72.6	66	27.4	3	42.9	4	57.1	0	0	3	100
Missed preschool	176	72.1	68	27.9	1	20.0	4	80.0	1	50.0	1	50.0
Trouble sleeping	175	72.9	65	26.4	2	22.2	7	77.8	1	50.0	1	50.0
Been irritable or frustrated	177	74.7	60	25.3	1	8.3	11	91.7	0	0	2	100
Avoided smiling or laughing	176	73.3	64	26.7	1	14.3	6	85.7	1	25.0	3	75.0
Avoided talking	176	72.1	68	27.9	1	20.0	4	80.0	1	50.0	1	50.0
Family impacts												
Felt upset	173	73.3	63	26.7	4	30.8	9	69.2	1	50.0	1	50.0
Felt guilty	169	75.1	56	24.9	8	33.3	16	66.7	1	50.0	1	50.0
Taken time off work	175	74.5	60	25.5	3	21.4	11	78.6	0	0	1	100
Had a financial impact on your family	177	72.8	66	27.8	1	14.3	6	85.7	0	0	1	100

309 parents/caregiver observations were recorded, of whom 187 had children with dmft = 0, 73 dmft > 0 and 58 unknown dmft

Regression analysis was performed on the complete dataset excluding all missing and unknown observations. As previously stated, initially, there were 340 children to be involved in the study. The final sample was reduced to 245 (after excluding 7.6% (26/340) of those who did not give consent and missing observations (69/340). This accounted for approximately 28% (95/340) not being available for statistical analysis. Thus as a result of this reduced data set, a number of categories had low numbers. Logistic models without a random effect were adequate in all three models as the estimated standard deviation for unexplained variation within each cluster had a value <0.0001. In the principle of parsimony, models with lowest AIC were utilised. With reference to the model examining family perspectives associated with dmft > 0, the AIC for the model, including random effects, was 284.14 and was 283.69 for the simplified model. Similarly, the model examining child related factors associated with dmft > 0, the AIC for the model including random effects was 274.87 and was 273.09 for the less complex model. Examining factors associated with ECOHIS > 0 resulted in the model including random effect having an AIC of 318.19 and the simplified model having an AIC of 316.19. The factors included in all models were free from multicollinearity as all adjusted GVIF values had values less than 2. Factors that could not be included for statistical analysis were ‘child avoided talking’ and ‘child being irritable or frustrated’, as these factors had excessively high adjusted GVIF values. The factor ‘child smiling’ was also omitted from the analysis due to zero observations for this factor with dmft = 0. Crude and adjusted ORs for children with dmft > 0 compared to those with dmft = 0 can be seen in Tables 4 and 5.

**Table 4:** Child factors associated with DMFT >0

	Crude OR	95% CI	Adjusted OR	95% CI
Child’s age				
3	1.0		1.0	
4	3.08	(1.59, 6.34)	3.61	(1.76, 7.95)
5	2.12	(0.74, 5.79)	2.26	(0.71, 6.83)
Child’s sex				
Male	1.0		1.0	
Female	0.89	(0.51, 1.54)	1.04	(0.57, 1.91)
Pain in the teeth, mouth or jaw				
No	1.0		----	
Yes	0.76	(0.31, 1.71)		
Difficulty drinking hot or cold drinks				
No	1.0		1.0	
Yes	12.55	(3.13, 83.85)	7.14	(1.36, 55.13)
Difficulty eating some foods				
No	1.0		1.0	
Yes	10.51	(3.16, 47.63)	8.29	(2.00, 43.49)
Difficulty pronouncing some words				
No	1.0		----	
Yes	3.42	(0.74, 17.76)		
Missed preschool				
No	1.0		----	
Yes	10.38	(1.5, 205.20)		
Trouble sleeping				
No	1.0		----	
Yes	9.76	(2.21, 64.62)		

Note: ‘Yes’ denotes occasionally/often/very often; ‘No’ denotes never/hardly ever

Model adjusted for other factors in the model

As confidence intervals are large, caution must be exercised when interpreting results

**Table 5:** Family factors associated with child’s DMFT > 0

	Crude OR	95% CI	Adjusted OR	95% CI
Parent/primary caregiver age				
< 25 years	0.61	(0.13, 2.16)	0.35	(0.05, 1.53)
25–34	1.12	(0.61, 2.07)	1.06	(0.56, 2.04)
35–44	1.0		1.0	
45+	0.31	(0.02, 1.81)	0.40	(0.02, 2.40)
Parent/guardian sex				
Male	1.0			
Female	1.66	(0.68, 4.67)	1.34	(0.53, 3.85)
Parent/primary caregiver highest level of education				
Primary or below	1.92	(0.71, 5.07)		
Secondary	1.38	(0.74, 2.60)		
Third level	1.0		----	
Other/unknown	0.54	(0.08, 2.25)		
Parent/guardian visits to dentist				
Never	1.49	(0.70, 3.07)		
1–2 per year	1.0		----	
Only when in pain	1.33	(0.59, 2.89)		
Other	1.11	(0.43, 2.67)		
Felt upset				
No	1.0		----	
Yes	6.13	(1.92, 23.30)		
Felt guilty				
No	1.0		1.0	
Yes	5.52	(2.28, 14.38)	3.50	(1.32, 9.60)
Taken time off work				
No	1.0		1.0	
Yes	9.29	(2.74, 42.45)	7.27	(1.76, 41.11)
Had a financial impact on your family				
No	1.0		----	
Yes	13.03	(2.05, 252.15)		
Type of pre-school				
Private school	1.0		----	
Government school	0.98	(0.54, 1.78)		

Note: ‘Yes’ denotes occasionally/often/very often; ‘No’ denotes never/hardly ever

Model adjusted for other factors in the model

As confidence intervals are large, caution must be exercised when interpreting results

Table 6 shows the crude and adjusted ORs for parents/primary caregivers with ECOHIS > 0 compared to those with ECOHIS = 0. Model evaluations indicate that the three models are adequate in terms of sensitivity, with AUC being estimated as 0.68, 0.64 and 0.74, respectively. However, as previously stated, these models were not developed for predictive purposes and caution must be exercised in model interpretation due to wide confidence intervals.

**Table 6:** Factors associated with ECOHIS > 0

	Crude OR	95% CI	Adjusted OR	95% CI
Parent/guardian age				
< 25 years	1.27	(0.43, 1.08)	1.59	(0.46, 5.44)
25–44	1.12	(0.41, 3.90)	1.11	(0.60, 2.07)
45–64	1.0		1.0	
65+	0.41	(0.05, 1.85)	0.55	(0.23, 1.28)
Parent/guardian sex				
Male	1.0		1.0	
Female	0.75	(0.35, 1.66)	0.55	(0.23, 1.28)
Parent/guardian highest level of education				
Primary or below	1.11	(0.44, 2.81)		
Secondary	0.82	(0.47, 1.43)		
Third level	1.0		----	
Other/unknown	0.54	(0.14, 1.79)		
Parent/guardian visits to dentist				
Never	0.68	(0.48, 0.95)		
1–2 per year	1.0		----	
Only when in pain	0.90	(0.42, 1.88)		
Other	1.70	(0.75, 3.89)		
Child’s age				
3	1.0		1.0	
4	1.63	(0.93, 2.88)	1.30	(0.70, 2.43)
5	1.37	(0.55, 3.35)	1.04	(0.38, 2.76)
Child’s sex				
Male	1.0		1.0	
Female	0.79	(0.47, 1.31)	0.83	(0.48, 1.47)
Child’s DMFT				
0	1.0		1.0	
1–3	2.68	(1.30, 5.64)	2.55	(1.19, 5.50)
> 4	8.58	(3.71, 22.45)	8.70	(3.54, 23.13)

Model adjusted for other factors in the model

In Model 1: child related factors associated with dmft > 0, statistically significant factors include child’s age, difficulty drinking hot or cold drinks, difficulty eating some foods, missing preschool and trouble sleeping (Table 4). Adjusting for the effects of other factors the odds for a child aged 4 years were greater for having dmft > 0 (in comparison to dmft = 0) than the odds for a child aged 3 years (OR 3.61; 95% CI (1.76, 6.83)). The odds for children who had difficulty drinking hot or cold drinks were greater for those with dmft > 0 than the odds for children who had no such difficulty (OR 7.14; 95% CI (1.36, 55.13)). Similarly, the odds of a dmft >0 were increased for children who have difficulty eating than the odds for children who have no difficulty eating (OR 8.29; 95% CI (2.00, 43.49)).

In Model 2: family perspectives associated with child’s dmft > 0, statistically significant factors include parent/primary caregiver feeling upset, guilty and having to take time off work (Table 5). Adjusting for the effects of other factors, the odds of parents/primary caregivers who felt guilty were greater for those with a child with a dmft > 0 in comparison to parents/primary caregivers who did not feel guilty (OR 3.50; 95% CI (1.32, 9.60)). Similarly, the odds of parents/primary caregivers who had to take time off work were greater with a child with dmft > 0, in comparison to parents/primary caregivers who did not have to take time off work (OR 7.27; 95% CI (1.76, 41.11)).

In Model 3: factors associated with ECOHIS > 0, a child’s dmft value were found to be statistically significantly related (Table 6). Adjusting for parental age/sex and child’s age and sex, model results indicate that the odds of parents/primary caregivers having ECOHIS >0 (in comparison to ECOHIS = 0), was increased when a child’s dmft was in the range 1–3 (OR 2.68; 95% CI (1.30, 5.64) or dmft > 4 (OR 8.58; 95% CI (3.71, 22.45)) compared with those whose child had a dmft = 0.

## Discussion

Overall, the frequency of oral health impacts for this Trinidadian sample was low for both Child and Family sections of the instrument, which is similar to data reported from the US [3]. As the majority of respondents had no impacts this may have resulted in a high ‘floor effect’. This can reduce the ability of the instrument to measure the interaction between the items in the child and family domains and OHRQoL. Unlike several other instruments, for the ECHOHIS the parent is asked to consider lifetime experience rather than the previous three months, to take account of lower disease levels in some populations.

The most frequent child impacts in this sample were similar to ECOHIS data from Australia, Canada, Iran, Hong Kong, Brazil and Turkey, which included English and non-English-speaking populations in developed and developing countries [11–16]. These main impacts were: *pain in the mouth*, *teeth or jaw*, *difficulty with eating some foods*, *drinking hot or cold beverages and being irritable or frustrated*. This suggests that OHRQoL impacts due to ECC are consistent across developed and developing countries. In a multiethnic population in a developing country, Malaysia, the main impacts were again similar, however, the prevalence of these impacts was much higher than in the present study [19]. This may have been due to the slightly older age groups (4–6 years) and differences in social/cultural backgrounds. These main impacts are consistent with symptoms from untreated dental caries in children and confirms the negative effect on quality of life that ECC can have in preschool children. Findings in the family section were also consistent with several other countries, where ECOHIS has been used, with *feeling guilty or upset* being the most common impacts (4,11,12,14,15,16). Interestingly, data from a Turkish study [16] differed from the present study findings, with most frequent family impacts being *financial* and *having to take time off work*, although this study was among a sample of older children with a higher severity of caries experience.

The issue of feelings of guilt about the oral health of their preschool aged child was explored by Carvalho et al. who defined guilt as “*a feeling that occurs when one assesses one’s specific action as a failure or when the particular action has led to failure*” [20]. The authors suggest that these feelings may be due to some parents having knowledge about prevention and dental care but are unable to act on it, effectively, with respect to their child.

In the present study the odds of having negative OHRQoL impacts for both the child and family were significantly associated with having visible caries experience. These odds increased with greater caries severity, indicating that families of children with untreated dental caries suffer the majority of the disease burden and should be prioritized for treatment and preventive care.

These findings highlight the need to develop oral health promotion strategies that support parents and caregivers and that go beyond merely increasing oral health knowledge. Changing behavior requires approaches that impart practical advice and enhance motivation, as well as developing coping skills, enabling families to overcome barriers to preventive dental care. In this regard, patient-centered counselling approaches and brief counselling techniques such as motivational interviewing (MI) have shown promise in relation to improving preschool children’s oral health [21, 22] and found to be an acceptable as part of health promotion for families of preschool children in Trinidad [23].

### Limitations of the study

1. This was a cross-sectional study from one education district and therefore limits the generalizability of the findings to the rest of Trinidad. However, the Caroni district does have a mixed of urban and rural population and a varied SES profile, similar to the national demographic profile.
2. Sampling was not stratified, which may have influenced the findings and masked differences by SES. Also, not all children under 5-years of age attend preschool and some of those children are likely to have had worse oral health than those in the sampling frame.
3. The findings for OHRQoL of preschool children are limited due to the use of proxy reports. Proxy reports on children’s oral health may underestimate the severity of oral health impacts.
4. Re-examination of children for intra examiner reliability took place on the same day, however, to avoid the bias due to memory of the initial examination, ideally, these should have been done on a return visit to the preschools.
5. Exclusion of very large preschools may have limited the representativeness of the sample.

## Conclusion

Although overall oral health impacts were low in this sample of preschool children, OHRQoL was found to be related to ECC. More impacts were found in children with greater severity of visible caries experience. The burden of dental disease and its impacts appears concentrated in a minority of young children, suggesting the need for strategies to address oral health in early childhood in Trinidad. Measuring the effect on OHRQoL in families with young children may enable prioritization and evaluation of interventions. Such interventions should support families in implementing positive dental care practices for their young children and include caries risk assessment, early establishment of the dental home and access to regular fluoride therapy for children at high risk for ECC, along with consistent information from dental health professionals, family physicians, pediatricians, community nurses, and preschool staff.

## Abbreviations

AAPD: American Academy of Paediatric Dentistry
ECC: Early childhood caries
ECOHIS: Early Childhood Oral Health Impact Scale
OHRQoL: Oral health related quality of life
